# Case report: Management and long-term ophthalmic sequelae of monogenean ocular infestation in cownose rays (*Rhinoptera bonasus*)

**DOI:** 10.3389/fvets.2024.1401141

**Published:** 2024-06-19

**Authors:** April Beatty, Anne Gemensky-Metzler, Georgina Newbold, Andrea C. Aplasca, Kathryn E. Seeley

**Affiliations:** ^1^Department of Clinical Sciences (Ophthalmology), The Ohio State University College of Veterinary Medicine, Columbus, OH, United States; ^2^The Maryland Zoo in Baltimore, Baltimore, MD, United States; ^3^Department of Animal Health, The Columbus Zoo and Aquarium, Powell, OH, United States

**Keywords:** cownose ray, capsalid monogenean, elasmobranch, veterinary ophthalmology, case report

## Abstract

Monogenean ectoparasitic flatworm infestations, particularly in closely confined populations, can result in severe epizootic disease that is often devastating and occasionally fatal. This case series describes a population of cownose rays (*Rhinoptera bonasus*) (*n* = 52) housed in an aquarium touch-tank exhibit that presented with severe ocular disease due to infestation with monogeneans, presumably *Benedeniella posterocolpa* of the Capsilidae family. The most severely affected individuals (*n* = 9), including several cases with bilaterally ruptured corneas, underwent serial examinations prior to and following treatment with praziquantel, systemic antibiotics, and corticosteroids. The entire population underwent frequent therapeutic water changes with a scheduled decrease in salinity, increase in temperature, and a series of seven praziquantel tank treatments. At the last follow up examination (3.75 years), the most common ocular findings were corneal fibrosis (18/18 eyes; 100%), cataract formation (13/18 eyes; 72.2%), synechia (8/18 eyes; 44.4%), and dyscoria (5/18 eyes; 27.8%). Despite severe corneal disease, including corneal rupture, all examined eyes (18/18; 100%) showed remarkable corneal remodeling and a largely clear visual axis. There are very few reports describing corneal disease in aquarium housed elasmobranchs, and no reports describe ophthalmic implications of monogenean infestation in these animals. This further underscores the importance of this case series in demonstrating the capacity for healing of elasmobranch eyes and can provide further guidance regarding prognosis and treatment in cases of severe corneal disease.

## 1 Introduction

Cownose rays (CNR), (*Rhinoptera bonasus*), are a species of cartilaginous fish belonging to the subclass, Elasmobranchii, alongside sharks, skates, and other ray species. Elasmobranchs are often included in large aquarium touch pools and CNR are the most commonly housed species in these exhibits (1). A documented condition affecting elasmobranchs, including CNR, is monogenean infestation. Monogeneans are host-specific parasitic flatworms that use hook-like structures, haptors, for attachment prior to feeding on mucus and epithelial cells resulting in a wide range of clinical implications (2). Clinical signs vary widely but commonly include skin and gill lesions, lethargy, and decreased appetite (1, 3, 4). The condition is often associated with high morbidity and mortality (2). While described in tilapia (*Oreochromis mossambicus*) (5), ocular involvement secondary to monogenean infestation has not been previously reported in elasmobranchs. There are a growing number of reports describing ocular disease in aquarium-housed CNR. In these studies, the etiology was not determined, however many rays were diagnosed with historic or current infestation of the skin and gills with *Benedeniella posterocolpa*, a monogenean of the Capsilidae family (6, 7). *Benedenellia posterocolpa* is considered the most likely causative agent of ocular infestation in this case series. These ectoparasites are reported to be large, oviparous monogeneans with a predilection for the ventral skin surface of CNR. Eggs are shed into the water column and can form an egg bank within the tank substrate. The 3–5 week life cycle (egg to egg), in addition to oviparity, renders treatment of tank infestations challenging (1). This represents the first report of confirmed monogenean ocular infestation in a population of aquarium-housed CNR, and includes information regarding treatment, long-term ocular implications, and prognosis.

## 2 Case description

### 2.1 Animals

**Figure 1:**
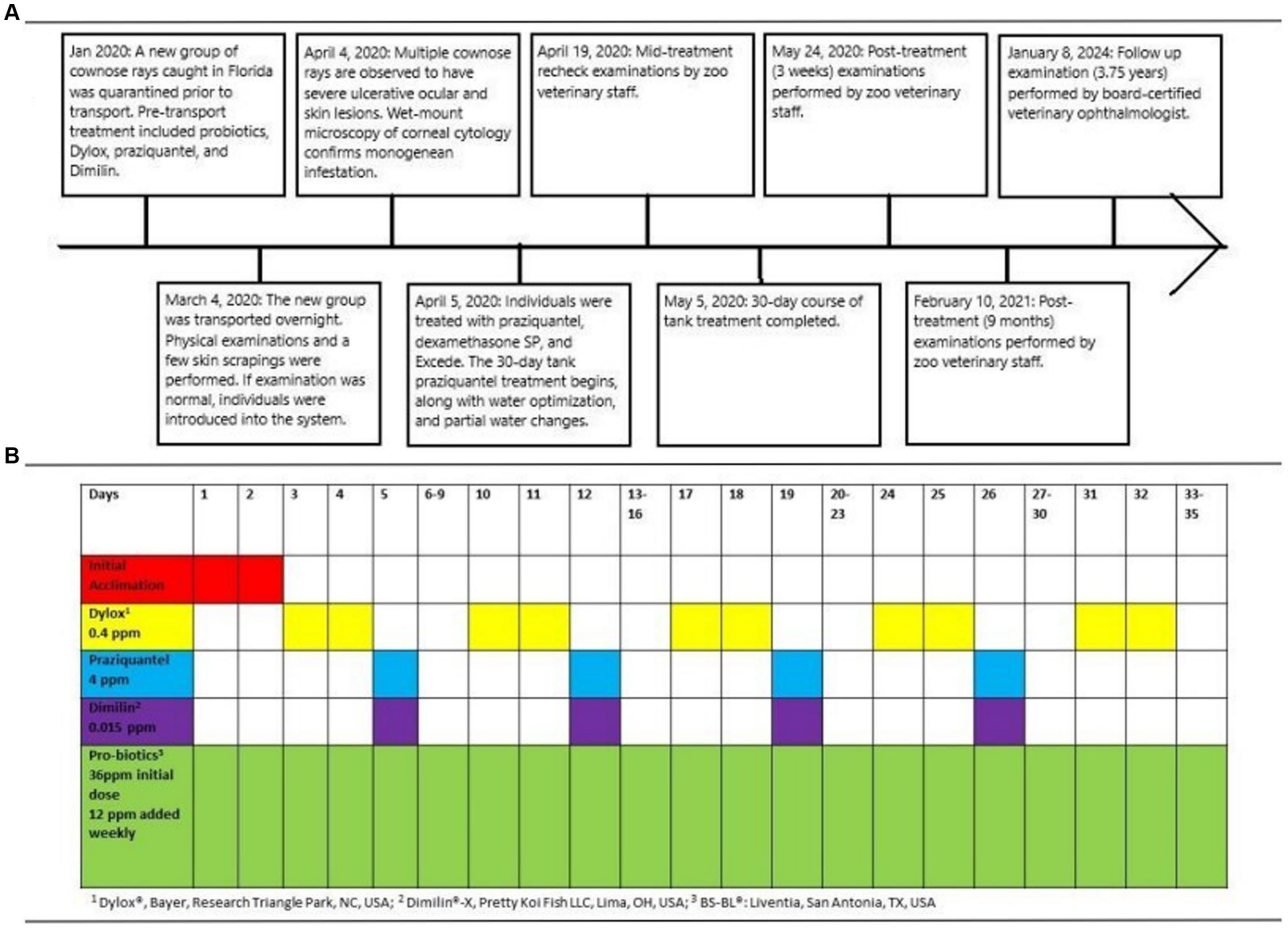
A timeline describing the history, diagnosis, treatment, and follow-up period of a confirmed ocular infestation by monogeneans in nine cownose rays (*Rhinoptera bonasus*) **(A)**, as well as a chronogram of treatments administered to incoming rays during the 35-day quarantine period prior to transport **(B)**.

Nine adult CNR (8 male, 1 female) housed in a touch tank exhibit were evaluated throughout this case series. Individuals were amongst a larger population of 52 CNR and 29 southern stingrays (*Hypanus americanus*) housed in a 9,000-gallon tank with sand substrate that is tightly controlled for temperature (75°F-80°F) and salinity (27-30 ppt). Saltwater is created in-house using commercially available sea salt mix (Instant Ocean® Sea Salt, United Pet Group, Inc., Cincinnati, OH, United States), and a 25% water change is performed weekly. Water quality is maintained by a life support system including a sand filter, protein skimmer and ozone. In Spring 2020, a wild caught group of 20 (16 male, 4 female) CNR from the Florida Keys, FL was introduced into the tank. All rays underwent quarantine treatments prior to transport (Figure 1). Transport occurred overnight in a temperature-controlled truck. There were no notable concerns during quarantine or transport. Individuals were examined by a veterinarian under manual restraint upon arrival and eight of 20 rays (40%) had skin scrapes performed. All examinations were within normal limits and no parasites were noted on wet mounts of the skin scrapes.

### 2.2 Clinical signs and diagnosis

**Figure 2:**
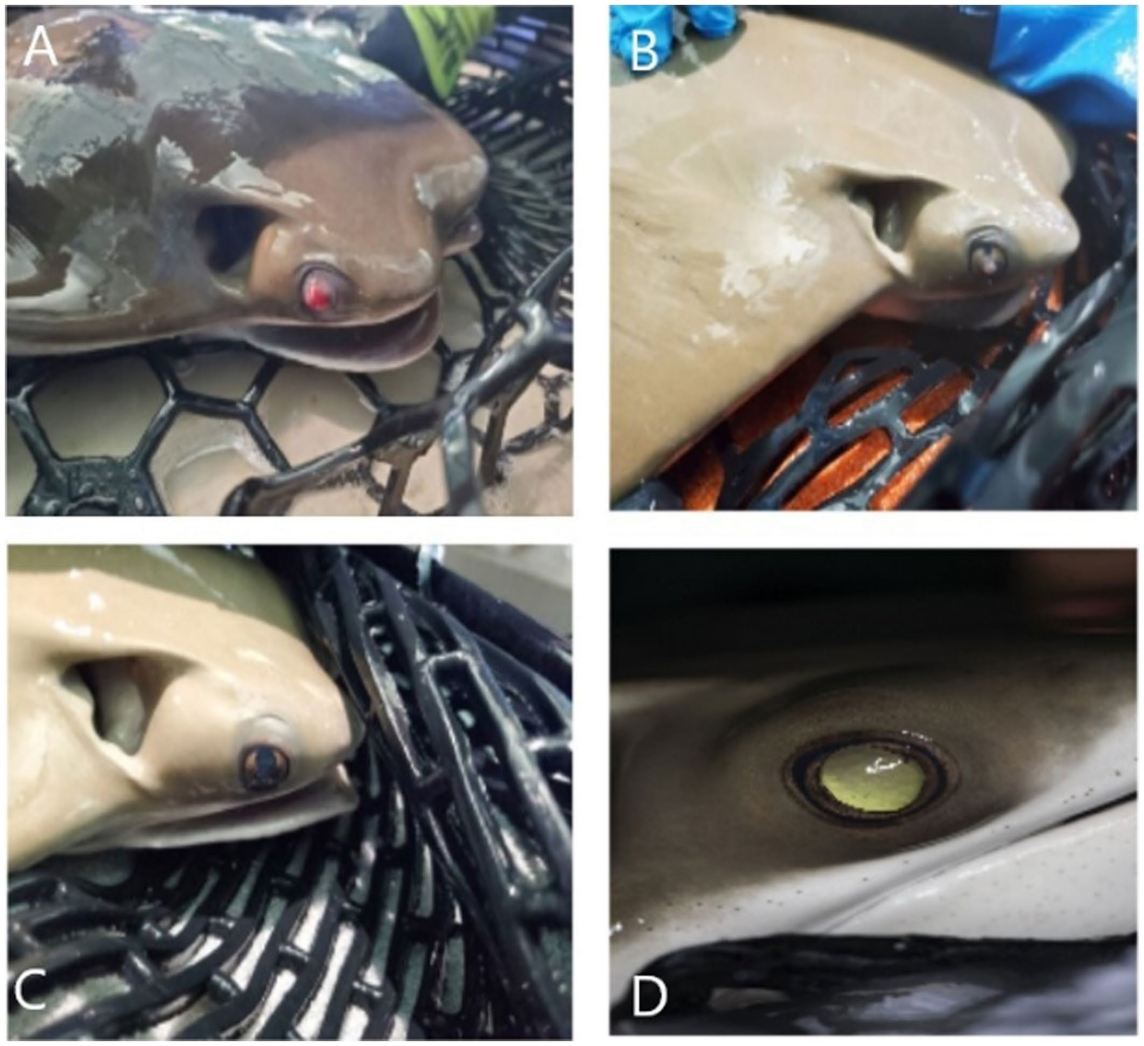
Photographs of the right eye of a cownose ray (*Rhinoptera bonasus*) at the time of confirmed monogenean ocular infestation **(A)** and at follow-up examinations halfway through treatment **(B)**, at completion of treatment **(C)**, and 3.75 years following completion of treatment **(D)**. **(A)** Axial corneal perforation with associated prolapsed hemorrhagic uveal tissue. **(B)** Axial conical fibrosis with severe dyscoria and presumed anterior synechia in location of previous perforation. **(C)** Improving corneal clarity and dyscoria. **(D)** Mild axial fibrosis, resolution of previous anterior synechia and dyscoria, focal posterior synechia at 1 o’clock position, incipient axial anterior cortical cataract (not pictured).

**Figure 3:**
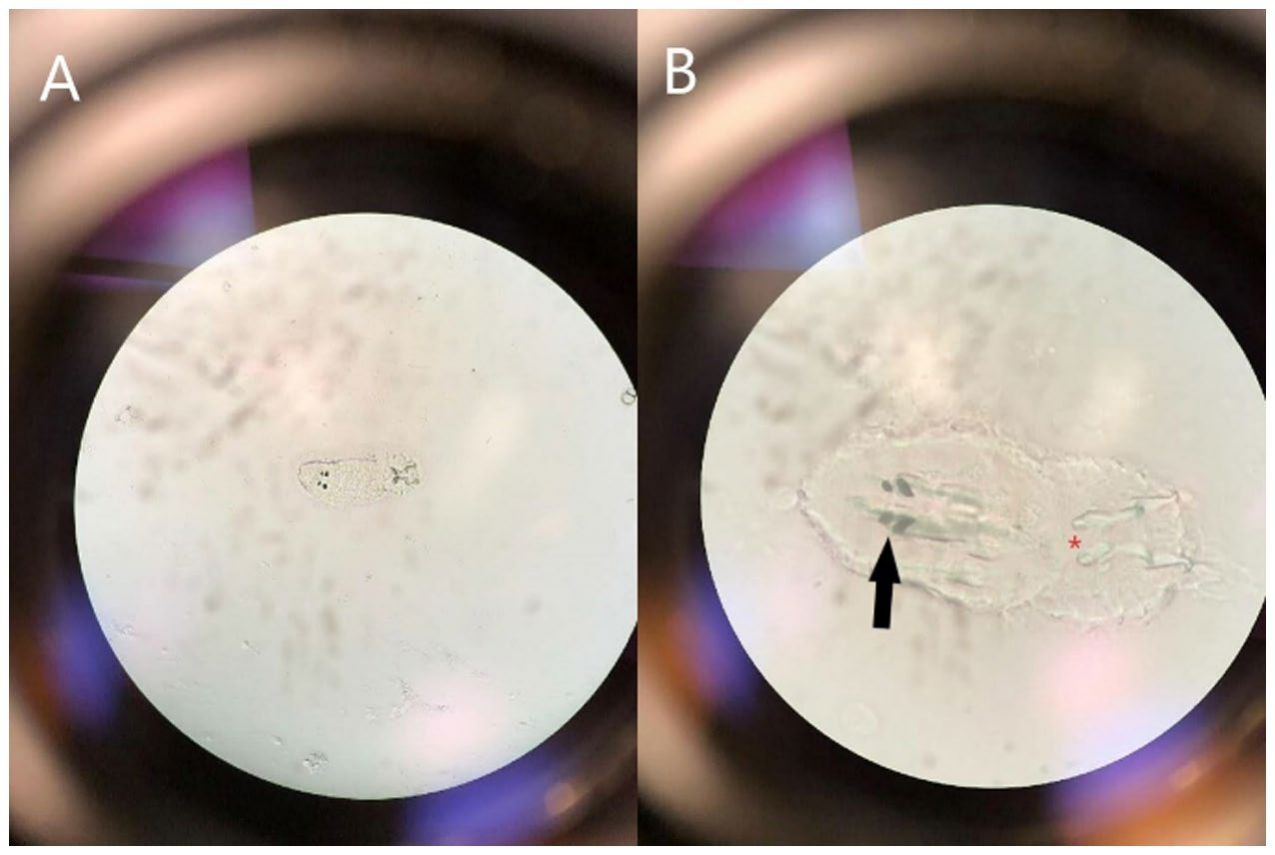
Photograph demonstrating the microscopic appearance of a ciliated oncomiracidium capsalid monogenoid, most likely *Benedeniella posterocolpa*, at 40X **(A)** and 100X **(B)** magnification collected via swab cytology from the corneal surface of a cownose ray (*Rhinoptera bonasus*). Distinguishing characteristics include two pairs of eye spots (black solid arrow) and two pairs of anchor hooks (red asterisk).

One month following introduction, numerous CNR were observed to have severe ocular and ulcerative skin lesions and were netted for examination. Eight of nine rays in this case series belonged to the recently introduced group. Examination by veterinary staff under manual restraint revealed corneal rupture in 14 eyes (14/18; 77.8%) (Figure 2). Of those not ruptured (4 eyes), four (4/4; 100%) were affected by corneal opacity resembling edema and one (1/4; 25%) had presumed ulceration and stromal loss. Fluorescein stain was not performed given logistical difficulty and severity of ocular changes. Of note, there were additional CNR affected, but only those with the most severe signs were netted for examination. All co-housed southern stingrays appeared clinically normal with no evidence of observable ocular or skin lesions. Given the high morbidity rate and recent introduction of new animals, an infectious condition was presumed. A sterile swab was used to collect corneal cytology from one individual and used for wet-mount preparation (8). Microscopy revealed an oncomiracidium, the ciliated larval form of a monogenean ectoparasite, with distinguishing characteristics of two pairs of eyespots and two pairs of anchor hooks (Figure 3) consistent with a diagnosis of capsalid monogenean infestation, most likely *Benedeniella posterocolpa* (1, 2, 9).

### 2.3 Treatment

Given the severity of clinical signs and fastidious nature of monogeneans, aggressive medical therapy was planned to target individuals, as well as the entire system. To reduce the burden of adult monogeneans quickly, individuals with visible ophthalmic lesions, including the nine in this study, were netted and placed in a praziquantel medicated bath [20 ppm, once, 30 min (praziquantel powder, Everything Aquatic, Medford, OR, United States)] the day following diagnosis (10–12). Supplemental oxygen was provided using an airstone and dissolved oxygen (DO) maintained between 90 and 100%. While in hand, individuals received dexamethasone sodium phosphate intramuscularly [0.25 mg/kg, once (Dexamethasone-SP, Bimeda-MTC Animal Health Inc., Ontario, Canada)] to reduce inflammation and ceftiofur crystalline free acid intramuscularly [80 mg, once (Excede, Zoetis Inc., Kalamazoo, MI, United States)] to prevent secondary bacterial infection. Following the bath, they were returned to the general population, but were isolated with baby gates for further monitoring. A water change was performed replacing at least 25% of the water, and a gradual concurrent decrease in salinity from 27 ppt to 18-22 ppt and increase in temperature from 77°F to 80°F was initiated. Water quality was measured daily, and animals were monitored to ensure tolerance of environmental changes. It was not feasible to treat all rays in the system with individualized bath treatments. Therefore, the entire system underwent episodic praziquantel administration in order to treat the less affected individuals and sand substrate, which could not be removed. Based on recommendations in the literature and consultation with colleagues, a lower dose of 8 ppm of praziquantel was dissolved into the system using rubber gloves and mesh stocking hose material every 4 days for a total of seven doses (28-day course) (12). Chemical and physical filtration were removed, and water quality was maintained by performing a 25% water change prior to each treatment cycle. Supplemental oxygen was added to the system to maintain DO. Praziquantel levels were tested to ensure therapeutic levels of >2 ppm were reached (Georgia Aquarium, Atlanta, GA, United States) (13). Immediately prior to redosing, the praziquantel level was 4.4 ppm and increased to 8.2 ppm following repeat dosing. Lastly, enrofloxacin [100 mg/g food (Baytril, Elanco US Inc., Shawness, KS, United States)] was added to the gel food for treatment and prevention of secondary bacterial infections and fed for a total of 30 days.

### 2.4 Follow up

**Figure 4:**
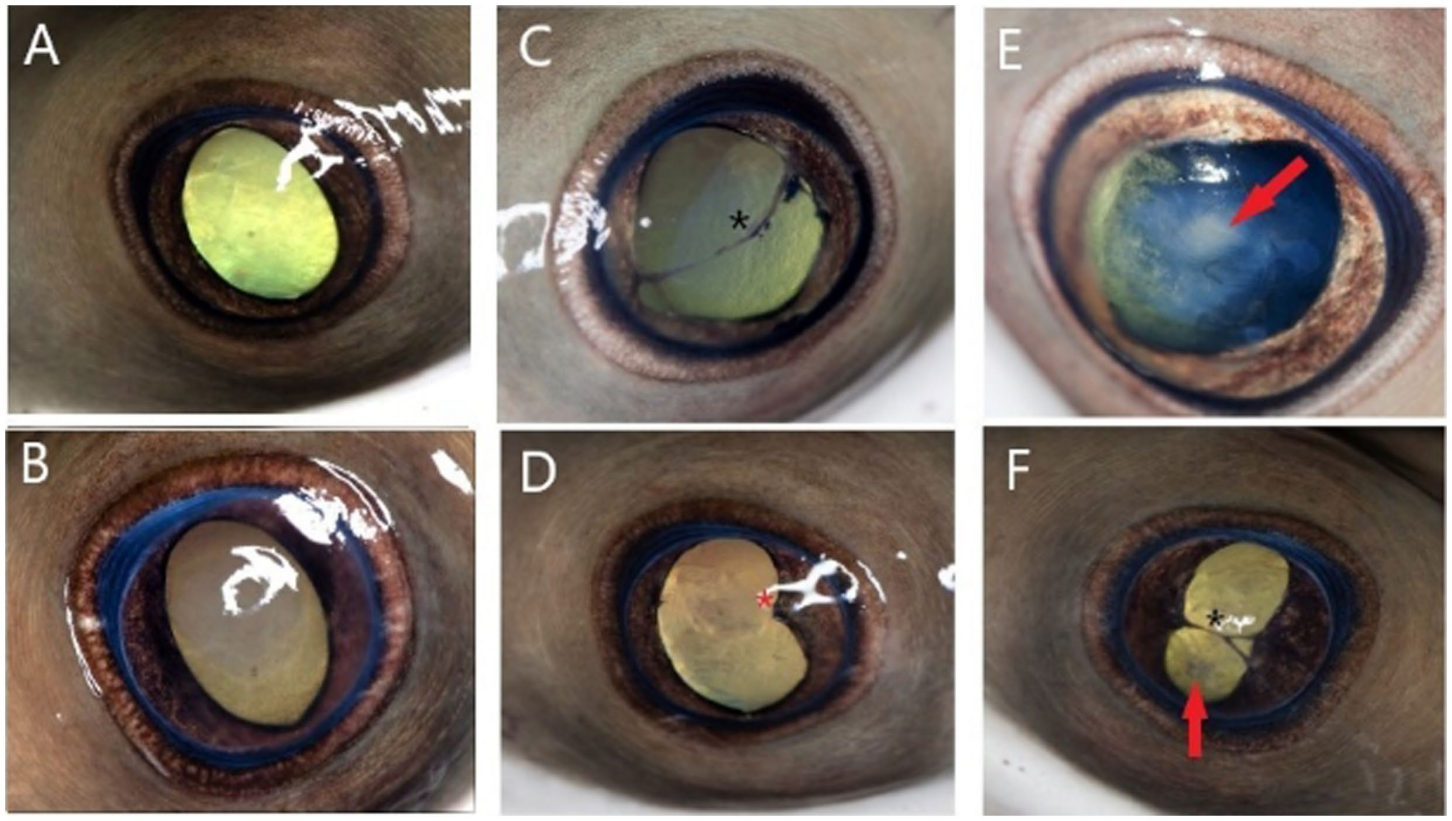
Photographs representing ophthalmic examination findings 3.75 years following monogenean ocular infestation in cownose rays (*Rhinoptera bonasus*). Findings are broadly classified as mild **(A,B)**, moderate **(C,D)**, and severe **(E,F)**. Examination findings are described as follows: **(A)** Mild axial corneal fibrosis, otherwise normal examination including ovoid pupil aperture; **(B)** Focal moderate axial corneal fibrosis, otherwise normal examination; **(C)** Moderate diffuse corneal fibrosis, slight dyscoria, multifocal endothelial pigment deposits, axial anterior synechia spanning the pupil aperture (black asterisk), incipient axial anterior cortical cataract (not pictured); **(D)** Moderate axial corneal fibrosis, dyscoria, focal posterior synechia nasally (red asterisk), axial incipient anterior cortical cataract (not pictured); **(E)** Severe axial corneal fibrosis, dyscoria, early immature axial anterior cortical cataract (red arrow); **(F)** Severe axial corneal fibrosis, severe dyscoria, anterior synechia spanning pupil aperture (black asterisk), incipient axial anterior cortical cataract (red arrow).

Examinations occurred 2 weeks following initiation of treatment, then 3 weeks, 9 months, and 3.75 years (45 months) after treatment was completed (Figure 1). Examinations 3 weeks following completion of treatment were performed without sedation by the zoo’s staff veterinarian, and all eyes (18/18; 100%) were documented to have healed corneal ulceration and signs of remodeling (Figure 2). The latest examinations were performed by veterinarians with specialty training in ophthalmology under sedation with 175 ppm tricaine methanesulfonate (Syncaine, Syndel, Ferndale, WA, United States) buffered 2:1 with sodium bicarbonate (Arm and Hammer baking soda, Princeton, NJ, United States). Depth of sedation was such that animals allowed handling but maintained voluntary respirations. All animals were recovered in non-medicated tank water and resumed swimming within 5 min. Ophthalmic examination included evaluation of the adnexa and anterior segment with a handheld slit-lamp biomicroscope (KOWA SL-17, Torrance, CA 90502, United States) and indirect ophthalmoscopy using a headset (Keeler, Malvern, PA, 19355, USA) and 30D lens (Volk Optical Inc., Mentor, OH, United States). Examination findings are listed in Supplementary Table 1. The most common findings were corneal fibrosis (18/18 eyes; 100%), cataract formation (13/18 eyes; 72.2%), synechia (8/18 eyes; 44.4%), and dyscoria (5/18 eyes; 27.8%) (Figure 4). Corneal fibrosis was graded as trace (1/18 eyes; 5.6%), mild (5/18 eyes; 27.8%), moderate (8/18 eyes; 44.4%), or severe (4/18 eyes; 22.2%). Cataracts were graded as incipient (10/13 eyes; 76.9%) or early immature (3/13 eyes; 23.1%), and the majority were in the axial anterior cortex. The fundus was successfully visualized in all 18 eyes and no posterior segment abnormalities were detected. All eyes (18/18; 100%) appeared functional, and all individuals continued to exhibit normal social and feeding behaviors.

## 3 Discussion

This report details the clinical presentation, treatment, and outcome of monogenean ocular infestation in aquarium-housed CNR. Normal anterior segment examination consists of a clear cornea, a relatively shallow anterior chamber devoid of aqueous flare or cellular infiltrate, an immobile vertically ovoid pupil aperture with individual variation in iris color from light tan to brown-black, and a clear lens. CNR lack eyelids, and their corneas are in constant contact with their aquatic environment (6). While ocular trauma has been reported (14), an infectious process was suspected given the percentage of individuals affected, recent introduction of new individuals, and absence of lesions in co-housed southern stingrays. Monogeneans are highly host specific which suggests why only CNR were affected (4). They have a direct life cycle increasing the risk of outbreak following introduction of infected animals because no intermediate host is necessary for infection to occur (2). Incoming individuals were likely infected prior to transport. It is possible they did not have substantial enough parasitic load to demonstrate clinical lesions as monogeneans can persist at low levels, and often do in wild settings, without clinical signs (2). Stress of transport may have exacerbated the condition, particularly given eight of nine animals were part of the incoming group. Additionally, Capsalidae species reproduce quickly, are oviparous, and eggs are resistant to treatment (10). Therefore, despite treatment with praziquantel during quarantine, it is possible a very small number of individuals or eggs could persist and quickly propagate to cause clinical disease (1, 3, 8).

Diagnosis of monogenean infestation is often made using wet-mount microscopy. Gross evaluation of morphologic features aid in determining which family of monogenean is present. Identification to the family level, specifically whether the reproductive strategy is oviparous or viviparous, is considered sufficient for management decisions. Further classification requires assessment of haptor and reproductive morphology or DNA sequencing, which was not performed in this study (8). Although species identification was not definitive, based on previous publications and presence of a capsalid oncomiracidium on cytology, *Benedeniella posterocolpa* was considered most likely (1). Once infestation with an oviparous monogenean was confirmed, a treatment strategy was determined. Individuals with severe lesions were considered likely to have the highest parasite burden and were therefore separated for individualized praziquantel medicated baths (2, 10). Group treatment aimed to eliminate all eggs and adult parasites within the environment given sand substrate could not be removed or replaced. The time required for maturation from eggs to adults is temperature dependent, lasting only a few days at higher temperatures. In contrast, cooler temperatures can result in an extended generation time up to 6 months (2). Therefore, water temperature was increased to promote quicker hatching of the eggs, and a 30-day treatment course of cyclical praziquantel administration planned every 4 days to target larvae hatching at varying time points. Praziquantel does not typically impact water quality, however can result in bacterial blooms that decrease DO and increase ammonia. Therefore, it is essential to monitor water quality during immersion treatment (8). Bacteria can begin to utilize praziquantel as a food source and cause degradation over time, necessitating higher doses to maintain therapeutic levels (8, 10). Since each system is unique and praziquantel is unpredictable, levels should be measured throughout treatment. This was not feasible in this case due to staffing and logistical limitations created by the outbreak of SARS-CoV-2. Freshwater dips have been described as a possible treatment in marine species, however a case report involving CNR suggests they may not systemically tolerate this dramatic decrease in salinity (15). Therefore, salinity was gradually decreased to a level deemed tolerable, but which may play a positive role in parasite elimination (16, 17). While a complete water change was not logistically possible, partial and frequent changes were performed throughout treatment.

To date, no reports have definitively confirmed ocular involvement of monogenean infestation in CNR (1, 2, 18). An abstract by Seyer et al. (7) describes ulcerative keratopathy in 11 captive CNR in which all had corneal lesions of varying severity. Interestingly, the Atlantic stingray (*Hypanus sabina*) examined in this report had no evidence of corneal disease. A species-specific etiology was suspected, but not determined. Additionally, Foote et al. (6) reports ophthalmic findings in 63 CNR from three separately housed groups. Nearly every ray (62/63) was diagnosed with ocular pathology including corneal abnormalities (opacity, ulceration, rupture), cataracts, and inactive intraocular changes (synechia). There was a significant association between active disease and which of the three facilities animals were housed. The aquarium with the most affected individuals had recently confirmed *Benedeniella posterocolpa* infestation on the skin of their CNR. A second aquarium had previously managed an outbreak of monogeneans confirmed using skin and gill scrapes. Multiple individuals were noted to have signs of active keratitis throughout the outbreak (6). While causation cannot be proven, it is probable the ocular changes described were related to a monogenean infestation with *Benedeniella posterocolpa*. These reports, in conjunction with this case series, support the use of corneal cytology and wet-mount microscopic evaluation in cases of keratitis in CNR.

Pathogenesis of corneal infestation and subsequent rupture in these cases is unknown. Monogeneans are found in mucus-laden environments and utilize haptors for epithelial attachment (1, 4). Corneal rupture may be a direct consequence of tissue destruction by monogeneans. It is also plausible the organism’s attachment to the cornea results in small ulcerations that become secondarily infected with bacteria, leading to keratomalacia and subsequent perforation (19). Alternatively, attachment may result in a foreign body sensation, and promote excess rubbing and self-trauma. Pathogenesis is likely multifactorial, and further research is warranted to determine the mechanism of corneal degradation and rupture in cases of monogenean ocular infestation as this information will guide future treatment strategies.

Long-term ophthalmic implications and prognosis following monogenean ocular infestation in CNR was previously unknown. Previous reports describe ocular disease secondary to monogenean infestation in tilapia (5), as well as other parasitic organisms such as copepods, (*Lepeophtheirus acutus*) in elasmobranchs (20). In tilapia, surviving individuals commonly had significant scarring and blindness (5), whereas elasmobranchs demonstrated significant healing and presumed functional globes following treatment similar to CNR described in this case series (20). While ocular lesions were noted in all 18 eyes, all were presumed visual at last examination given a relatively clear visual axis and normal fundus. Aquatic species rely on spherical lenses as the major refractive organ of the eye (21), likely rendering residual corneal opacities and fibrosis (18/18 eyes; 100%) in these individuals even less significant in terms of impacting visual acuity. While cataract was relatively common (13/18 eyes; 72.2%), the majority were incipient and did not impede visualization of the posterior segment. Elasmobranch corneas are considered the most primitive of all vertebrates and contain sutural fibers in the stroma as an adaptation to prevent swelling in cases of epithelial disruption (22). This distinct structural difference, in addition to documented wound-healing capabilities of elasmobranchs, likely contributes to exceptional healing following severe ocular disease, including corneal perforation (23, 24). Corneal angiogenesis is a nonspecific response to inflammation in most species (25). Interestingly, corneas of CNR remained relatively avascular throughout follow-up suggesting either an alternative response to keratitis that does not involve significant angiogenesis or rapid regression of vessels to restore translucency. While it may be tempting to consider enucleation or euthanasia in cases of advanced ocular disease, this case series suggests medical therapy may be successful for both healing and preserving long-term vision in CNR.

A limitation of this case series is that baseline and subsequent ophthalmic exams were only performed in nine CNR. Additional individuals were documented to have inactive ocular lesions on future examinations, but were not examined at initial diagnosis. This may overestimate severity of lesions as those evaluated prior to treatment were those with the most observable lesions. Furthermore, rays not examined at initial diagnosis did not receive individual treatment but were subject to treatments of the entire system making interpretation of the necessity of individualized treatments difficult. An additional limitation is that the species of monogenean was not identified. While family classification is sufficient for guiding treatment and *Benedeniella posterocolpa* is considered most likely, interpretation about which species of monogeneans plays a more significant role in ocular disease cannot be definitively stated. Lastly, interpretation of functional vision was based on presence of a clear visual axis, absence of posterior segment abnormalities, and normal behavior. Future investigation may include a prospective case series evaluating all individuals, species identification, pathogenesis, and objective vision assessment to further understand this complex condition of elasmobranchs.

This report is the first to describe clinical implications associated with confirmed ocular monogenean infestation in CNR. Signs of keratitis appear to be associated with monogenean infestation and can be as severe as corneal perforation. Corneal cytology and wet-mount microscopy should be considered in cases of ocular disease in elasmobranch species. Treatment of both individuals and the system using praziquantel, antibiotics, anti-inflammatories, water changes, and optimization of water salinity and temperature may be a successful strategy for eliminating parasites. Despite the severe presentation, CNR demonstrated exceptional healing capacity of ocular structures and all eyes were presumed to retain vision 3.75 years following diagnosis supporting the notion that aggressive medical therapy should be considered in cases of ocular monogenean infestation.

## Data availability statement

The original contributions presented in the study are included in the article/Supplementary material, further inquiries can be directed to the corresponding author.

## Ethics statement

The animal study was approved by the Columbus Zoo and Aquarium Research Committee. The study was conducted in accordance with the local legislation and institutional requirements.

## Author contributions

AB: Conceptualization, Data curation, Formal analysis, Investigation, Writing – original draft, Writing – review & editing. AM: Conceptualization, Investigation, Methodology, Supervision, Writing – original draft, Writing – review & editing. GN: Writing – original draft, Writing – review & editing. AA: Data curation, Investigation, Writing – original draft, Writing – review & editing. KS: Conceptualization, Data curation, Investigation, Methodology, Resources, Supervision, Writing – original draft, Writing – review & editing.
